# A History of Low-Dose Ethanol Shifts the Role of Ventral Hippocampus during Reward Seeking in Male Mice

**DOI:** 10.1523/ENEURO.0087-23.2023

**Published:** 2023-05-15

**Authors:** Kathleen G. Bryant, Mitchell A. Nothem, Lauren A. Buck, Binay Singh, Sana Amin, Christina M. Curran-Alfaro, Jacqueline M. Barker

**Affiliations:** Department of Pharmacology and Physiology, Drexel University College of Medicine, Philadelphia, PA 19102

**Keywords:** electrophysiology, ethanol, hippocampus, motivation, operant, optogenetics

## Abstract

Although casual drinkers are a majority of the alcohol drinking population, understanding of the long-term effects of chronic exposure to lower levels of alcohol is limited. Chronic exposure to lower doses of ethanol may facilitate the development of alcohol use disorders, potentially because of ethanol effects on reward learning and motivation. Indeed, our previously published findings showed that chronic low-dose ethanol exposure enhanced motivation for sucrose in male, but not female, mice. As the ventral hippocampus (vHPC) is sensitive to disruption by higher doses of chronic ethanol and tracks reward-related information, we hypothesized that this region is impacted by low-dose ethanol and, further, that manipulating vHPC activity would alter reward motivation. *In vivo* electrophysiological recordings of vHPC population neural activity during progressive ratio testing revealed that vHPC activity was suppressed in the period immediately after reward seeking (lever press) in ethanol-naive controls, whereas suppression of vHPC activity anticipated reward seeking in ethanol-exposed mice. In both ethanol-naive and exposed mice, vHPC activity was suppressed before a reward magazine entry. Temporally selective inhibition of vHPC using optogenetics increased motivation for sucrose in ethanol-naive controls, but not in ethanol-exposed mice. Further, regardless of exposure history, vHPC inhibition promoted checking of the reward magazine, indicating a role for vHPC in reward tracking. There was no effect of chemogenetic inhibition of the vHPC either during training or testing on sucrose reward motivation. These results reveal novel ethanol-induced alterations in vHPC neural activity that shift how vHPC activity is able to regulate reward seeking.

## Significance Statement

A large portion of the population consumes alcohol at levels that are subthreshold for an alcohol use disorder. Low-dose ethanol exposure could help convey susceptibility to alcohol use disorders by disrupting activity in brain regions that are important in reward seeking and motivation like the ventral hippocampus (vHPC). Here, we found that a history of low-dose ethanol exposure shifted ventral hippocampus encoding of actions in mice, such that it altered the role of ventral hippocampus in reward seeking. These findings further our understanding of the impacts of low-dose ethanol exposure on motivated behavior and reveal ethanol-induced modulations in neural correlates of reward motivation.

## Introduction

A majority of adults who consume alcohol drink at levels that do not meet diagnostic criteria for an alcohol use disorder (SAMHSA, 2021). Despite the high prevalence of low-dose ethanol consumption, the effect of low-dose ethanol on brain function and behavior remains poorly understood. Recently published data from our lab found that a history of chronic low-dose ethanol exposure increases motivation for sucrose in male, but not female, mice (Bryant et al., 2022). Further, this increased motivation in males was associated with reduced reward tracking behavior, suggesting that low-dose ethanol exposure produced long-term changes in reward seeking behavior and behavioral strategy selection. However, the neurobiological changes resulting from chronic low-dose ethanol exposure that support shifts in reward motivation and tracking are largely uncharacterized.

The ventral hippocampus (vHPC) is increasingly considered to be a critical contributor to reward learning and reward seeking behavior (Ito et al., 2008; Schumacher et al., 2016; Riaz et al., 2017; Barfield and Gourley, 2019). It has been theorized that suppressed vHPC activity is necessary for the performance of flexible actions (Gray, 1977; Bryant and Barker, 2020). Indeed, lesioning or inhibition of the vHPC promotes goal-directed and approach-related behaviors during reward seeking (Kjelstrup et al., 2002; D. R. Bach et al., 2014; Ito and Lee, 2016; Schumacher et al., 2016) and chemogenetic suppression of vHPC activity can restore goal-directed behavior (Barker et al., 2019). Thus, changes in vHPC activity surrounding behavioral events could underlie differences in reward seeking through dysregulation of downstream targets.

The vHPC is particularly sensitive to perturbation by ethanol exposure. Chronic ethanol consumption produces significantly greater pyramidal neuron loss in the ventral versus dorsal hippocampus (Lescaudron and Verna, 1985). Recent studies have shown that chronic intermittent ethanol-exposed (175–225 mg/dl) mice and rats exhibit reductions in vHPC calcium activity and increases in vHPC synaptic excitability that may drive further ethanol seeking behavior (Ewin et al., 2019; E. C. Bach et al., 2021; Griffin et al., 2023). To our knowledge, all previous research into chronic ethanol effects on the vHPC has investigated changes in vHPC in models of ethanol dependence, which produces changes different from lower levels of exposure or even binge alone (Crabbe et al., 2011; Becker, 2013). Given our recent finding that chronic low-dose ethanol alters reward-seeking strategies, we hypothesized that ethanol-driven shifts in behavioral strategy would be accompanied by shifts in vHPC neural activity patterns given its role in reward seeking and sensitivity to ethanol-induced perturbation. This study thus integrated *in vivo* recordings of vHPC population neural activity during motivated behavior to characterize low-dose ethanol effects on vHPC activity and closed-loop optogenetic inhibition of vHPC activity to demonstrate a direct role of vHPC in regulation of sucrose reward motivation and behavioral strategy selection.

## Materials and Methods

### Subjects

Adult male C57BL/6J mice (nine weeks of age; *n* = 98) from The Jackson Laboratory were used in these studies in accordance with the Drexel University Institutional Animal Care and Use Committee guidelines. The goal of this study was to investigate the neural correlates underlying low-dose ethanol-induced disruptions in motivated reward seeking, which were observed only in male, but not female, mice (Bryant et al., 2022). Thus, only male mice were used in this study. Mice were housed in a vivarium with a standard 12/12 h light/dark cycle and were acclimated to the animal facility for one week before beginning any experiments. A subset of mice (*n* = 24) underwent stereotaxic surgery with a retrograde pAAV_rg_-hSyn-EGFP (Addgene plasmid #50465, RRID: Addgene_50465) targeting the nucleus accumbens shell (anterior/poster (AP) +1.5 mm, medial/lateral (ML) +0.6 mm, dorsal/ventral (DV) −4.7 mm) before beginning behavioral experiments for later tract tracing. Other mice were assigned to receive stereotaxic surgery for electrode array implantation, optic fiber implantation, or virus injection as described below. Following recovery from surgery or following the acclimation period, mice were food restricted to ∼90% of their *ad libitum* weight and maintained at that weight for the length of the experiments. All mice, except for electrode-implanted and optic fiber-implanted mice, were group housed for the duration of the study.

### Operant set-up

All operant training occurred in standard Med Associates operant boxes for mice in sound attenuating chambers. The right wall of the operant chamber was equipped with two retractable levers on either side of a recessed magazine where liquid reinforcer could be delivered into a small receptacle with a syringe pump. The left wall of the chamber was equipped with five nose poke holes and hole lights, but they were not used for the current studies. The back wall and the ceiling of the chamber were clear Plexiglas, and a standard metal bar floor was used. A house light that turned on at the start of the session and remained on for the session length was fitted above the magazine. No discrete cues were ever presented with reward delivery or responding.

For experiments in electrode-implanted mice the exact same operant setup was used, with the addition of minor changes to reduce electrical noise and allow for headstage tethering during behavior. The left wall was blocked with clear Plexiglas to reduce electrical noise. The clear Plexiglas ceiling had a hole for the headstage cable. The normal recessed magazine was replaced with a taller magazine to accommodate the headcap and cable setup. For optic fiber-implanted mice, the set-up was the same except a taller, wider magazine (Med-Associates standard “rat” operant box sizing) was used to accommodate the stiffer optic fiber cables used.

### Instrumental training

Before beginning operant training, mice underwent two, 15-min sessions of magazine training where a 10% liquid sucrose solution (20 μl) was delivered into the magazine every 60 s to train the mice on the reward delivery mechanism and to acclimate them to the 10% sucrose reward. After two sessions of magazine training, mice began training on a fixed ratio 1 (FR1) schedule where every lever press resulted in reward delivery. The mice were trained to respond on two separate levers that were presented consecutively during the behavioral session, such that one lever (e.g., right lever) was presented and accessible for the first half of the session, then that lever retracted and the other lever (e.g., left lever) was accessible for the rest of the session. The order of which lever was accessible first alternated every day for each mouse and was counterbalanced across all groups and conditions.

Presses on either lever were reinforced with sucrose on a FR1 schedule until stable responding on both levers (>15 lever presses on each lever for at least 3 d) was acquired. Mice that never acquired stable responding were excluded (*n* = 7). Once stable responding was acquired, the schedule of reinforcement for each lever diverged such that the left lever was reinforced on a random interval (RI) schedule and the right lever was reinforced on a variable ratio (VR) schedule. These schedules were chosen because they have been shown to promote differing behavioral strategies, with RI schedules generally promoting inflexible, habitual behavior while VR schedules maintain flexible, goal-directed action (Dickinson et al., 1983; Gremel and Costa, 2013; Barker et al., 2017). The RI schedule used is a time-based schedule that reinforces the first lever press after a randomly determined interval averaging 30 s for RI30 and 60 s for RI60. The VR schedule used is an effort-based schedule that reinforces every Y^th^ lever press, where Y averages 5 for VR5 and 8 for VR8. Mice underwent 3 d of RI30/VR5 training followed by 3 d of RI60/VR8 training before testing. Only data from the left lever (FR1 to RI30 to RI60) are being shown and analyzed for the purposes of this manuscript.

### Ethanol exposure

Mice were randomly assigned to receive injections of saline or low-dose ethanol (0.5 g/kg, i.p.) 1 h after the start of the behavioral session, as our previous findings identified this time as a critical period for ethanol-induced changes in male mice (Bryant et al., 2022). Injections followed each training session, beginning at the first day of FR1 through the final day of RI60 training. No injections were given after the final training day during motivation testing or retraining; thus, mice were not exposed to ethanol or in acute withdrawal when motivation was assessed. Injections occurred in the home cage in the animal housing facility and mice were given their daily food allotment after behavior every day, before injections occurred. Following completion of all behavioral experiments, a subset of mice received low-dose ethanol (0.5 g/kg, 7% v/v, i.p.) injections and blood was collected using submandibular bleeds either 30 min or 60 min after acute injection. The blood samples were then processed and analyzed for blood ethanol concentration (BEC) using an Analox system.

### Progressive ratio testing and analysis

In order to determine the effort mice were willing to exert for sucrose reward, mice were tested on an arithmetic progressive ratio 4 (PR) schedule, where the number of lever presses required for each subsequent reinforcer delivery was increased by four each time a reinforcer was delivered (1, 5, 9, 13, 17, etc.). The maximum ratio reached was determined by either the animal’s “break point,” the lever press ratio at which they stopped pressing for 5 min, or by the maximum ratio reached within the maximum session length. The maximum session length was 2 h for all electrode-implanted mice, 4 h for all optic fiber-implanted mice, and 8 h for all nontethered (e.g., Designer Receptors Exclusively Activated by Designer Drugs; DREADD) mice. These different session lengths were chosen because of the effects of tethering on behavior, and the average session length for mice in each condition to reach their break points for each experiment. Most mice (*n* = 109 PR sessions; *n* = 67 mice) reached their break point within the confines of the session length. Mice that did not reach break points within the time limit were still included in the analysis using the maximum ratio reached before session termination and this did not vary by condition [(electrode implanted mice *n* = 2 (1 ethanol, 1 saline); optic fiber implanted mice *n* = 4 (3 unpaired sessions, 1 paired session); DREADD mice *n* = 4 (2 GFP + clozapine-n-oxide (CNO), 1 DREADD + CNO, and 1 DREADD + saline)]. PR testing began one to three weeks following the completion of behavioral training. One additional day of RI60/VR8 retraining was given before PR testing because of the large average length of time from the last training session to the first PR test day. No ethanol was administered at the retraining sessions. A subset of mice were tested for habits using a contingency degradation test after behavioral training but before beginning testing on the PR. Data from those additional tests are being excluded for the purposes of this manuscript and behavioral testing on these other paradigms did not impact behavioral patterns on the PR test.

Additional analyses of the PR data, including magazine checking, were conducted by extracting and analyzing Med Associates time-stamped PR data using MATLAB code generated by our lab and as described previously (Bryant et al., 2022; https://github.com/bsingh0110/Progressive-Ratio-Analysis-).

### Multielectrode array implantation

A subset of male C57BL/6J mice (*n* = 14) were unilaterally implanted with 16-channel tungsten microwire arrays purchased from Innovative Neurophysiology with wires targeting vHPC (AP −2.9 to −3.8 mm, ML +3.0 to +3.2 mm, DV −4.0 mm) in an eight by two arrangement (35-µm diameter, 150-µm spacing). Surgeries were performed under isoflurane anesthesia and implants were secured to the skull using Metabond (Parkell) and dental cement. Two mice were excluded because of problems with the implant postoperatively. At the end of the experiments, mice were perfused using a 4% paraformaldehyde in phosphate buffered saline solution. A glial scar including astrocytes progressively forms around the wire following implantation. Wire placement was confirmed by immunohistochemical staining for glial fibrillary acidic protein (GFAP; Sigma-Aldrich, catalog #G9269, RRID: AB_477035), an astrocyte cytoskeletal protein, which enables visualization of the wire track and tips as has been described previously (Dickey et al., 2009; Falcone et al., 2018; Giacometti et al., 2020; Pflüger et al., 2020).

### Neurophysiological data collection and analysis

To investigate vHPC activity during motivated behavior, following recovery from surgery, mice began behavioral training and food restriction as described earlier. During key behavioral sessions, including PR testing, mice were connected to a Plexon OmniPlex system and vHPC neural activity was recorded. Mice were connected via a Plexon head stage (HST/16D) and digital head stage cable (HSC/DHSC1). The OmniPlex system directly integrates Med Associates outputs via TTL pulses enabling time-locked integration of behavioral and electrophysiological measures. All behavioral outputs (lever press, reinforcer delivery, and magazine entry) were recorded as distinct TTL outputs on the system, in addition to a TTL pulse that signaled the start of the session. For implanted mice, the PR test session was truncated from the normal maximum session length of 8 to 2 h. This shorter session length was chosen because a majority of the behavioral changes were observed within the first 2 h of the session. Most tethered mice (*n* = 10) reached break points within the 2-h window, as the tether and recording set-up impacted response rates.

### Spike sorting and electrophysiological analysis

Before the start of recorded behavioral sessions, headstage cables were connected and mice were able to freely roam their homecage while online sorting and referencing were completed using PlexControl software (Plexon). Signals were digitized at 40 kHz using a Plexon Omniplex system and filtered with a band pass filter from 300 to 6000 Hz. Spikes were detected by being at least four sigmas from threshold and were discriminated via examination of various features and principal component views of the waveforms. Initial online sorting was saved to a hard disk and filtered for initial processing using a high band and low band filter, then refined later using Plexon Offline Sorter. Since electrophysiological recordings mainly constituted multiunit activity and single units cannot be sorted on some channels with high confidence, single unit analysis was not performed. Still, a large number of “units” were recorded for each group (saline *n* = 149, average of 30 per animal; ethanol *n* = 219, average of 36 per animal). Instead, spiking activity from each channel was averaged across each animal and channels with no single or multiunit activity were excluded from analysis. Perievent time histograms were generated using NeuroExplorer software (Plexon) by binning spikes (100-ms bins) around each behavioral event (magazine entries or lever presses). Raw z-scored binned spike data were used for all statistical analyses, but data were smoothed using a Gaussian filter (three bins) for graphing. Perievent histograms were z-scored using all spikes within the behavioral windows: −1.0 to 0.5 s for lever presses and −4.0 to 2.0 s for magazine entries.

### Optogenetic surgery and behavior

A subset of male C57BL/6J mice (*n* = 42) underwent bilateral microinjections of either a GFP- (pAAV1-CaMKIIa-EGFP; 0.5 µl; Addgene plasmid #50469; RRID:Addgene_50469) or ArchT-expressing (pAAV8-CaMKII-ArchT-GFP; 0.5 µl; Addgene plasmid #99039; RRID:Addgene_99039) virus targeting the ventral hippocampus (vHPC; AP −3.2 mm, ML ±2.8 mm, DV −4.0 mm). The pAAV-CamKII-ArchT-GFP (PV2527) was a gift from Edward Boyden. Optic fibers (Thor Labs #CFML12U-20 cut to 3.8 mm) were implanted just above the virus injection (DV −3.8 mm). One mouse in the ethanol-exposure group was excluded for incorrect viral and optic fiber placements.

The optogenetic experiments used a set-up built from Thor Labs equipment. LED light was delivered to the optic fiber using a 565 nm LED laser (Thor Labs #M565F3) connected to a rotary (Thor Labs #M76L01 and #RJ1) and bifurcated optic patch cable (Thor Labs #BFYL1LF01) which interfaced with the implanted fiber using a ceramic sleeve (Thor Labs #ADAL1). The LED laser was driven by outputs from a MED-PC program which connected to the LED driver (LEDD1B) through a 28V DC to TTL adapter from Med Associates (SG-231). The LED light power that was targeted and achieved at the tip of each bifurcated cable was ∼1.5–3.0 mW (average light power was 2.5 mW).

### Optogenetic modulation of ventral hippocampus activity

To determine whether optogenetic inhibition of vHPC surrounding a lever press could shift break points on the PR, mice recovered from surgery for one week, and then were trained and exposed to saline or ethanol as described above. Following all training, mice were tested on the PR test, where optogenetic manipulations occurred. All optogenetic manipulations occurred in either a paired or unpaired condition. In the paired condition, the LED light was paired to a lever press such that it was turned on for 0.5 s directly following a lever press. In the unpaired condition, the LED light was unpaired from the lever press such that it occurred at a fixed time interval based on each animal’s baseline response rates on the tethered RI60/VR8 session day before the PR test. In this condition, it was further programed so that the LED light did not turn on during the 2-s window following a lever press. All mice except for a subset of the GFP mice (*n* = 3) were tested on both conditions. The order of which condition was tested was first counterbalanced across all groups. A tethered RI60/VR8 session where the mice were plugged in, but no LED light was ever on, was given the day before each PR test to retrain them on the normal schedule/action-outcome relationship, and to prevent extinction from consecutive PR tests.

### Chemogenetic inhibition of the ventral hippocampus

To determine whether chronic inhibition of the vHPC during training altered reward seeking behavior, a subset of mice (*n* = 24) underwent stereotaxic surgery for a bilateral injection of either a DREADD receptor- (pAAV-CaMKIIa-hM4D(Gi)-mCherry; 0.2 µl; Addgene plasmid #50477; RRID:Addgene_5477) or GFP-expressing (pAAV1-CaMKIIa-EGFP; 0.2 µl; Addgene plasmid #50469; RRID:Addgene_50469) virus targeting the vHPC (same coordinates as listed above). The pAAV-CaMKIIa-hM4D(Gi)-mCherry was a gift from Bryan Roth. Beginning three weeks after surgery to allow for virus expression, mice were trained and tested as described above, except mice were not exposed to any saline or ethanol. Instead, starting on the first day of FR1 training and continuing through the final day of RI60/VR8 training, mice were given either saline or clozapine-n-oxide (CNO; 2.0 mg/kg, i.p.) 30 min before beginning each training session. Similar to the ethanol exposure model, there were no more injections of CNO or saline following the final training day or during PR testing. Two mice were excluded from analysis, one DREADD + saline mouse was removed from the study during the postoperative period, and one DREADD + CNO mouse had incorrect viral injection placements. A subset of mice from the DREADD + Saline group (*n* = 7) received injection of CNO or saline in a second PR test to determine the effects of vHPC inhibition during PR testing on break points.

### Experimental design and statistical analysis

Experimental design, animal numbers, and exclusions are as listed above for each experiment. Animal numbers of ∼10 per group for nonimplanted mice and six per group for implanted mice were targeted based on previous effect size analyses on behavioral experiments in our lab. When possible, within-subjects designs were used (e.g., vHPC activity bins, optogenetic data). GraphPad PRISM was used for all statistical analyses and a statistical table has been included (Table 1). BEC data and some of the optogenetic comparisons were analyzed using an unpaired *t* test. An ANOVA test was performed for behavioral outputs and population recording data. Repeated measures ANOVA (rmANOVA) was used when appropriate in analyses that involved within-subjects and between-subjects comparisons (behavioral training analysis, recording data analysis, etc.). Šídák’s and Tukey’s corrections were used for *post hoc* analyses. Correlational analyses were performed using linear regression.

**Table 1:** Table of all statistical and estimation analyses in the manuscript

	Data structure	Type of test	Power
a	Normal	Unpaired *t* test	0.8399
b	Normal	Unpaired *t* test	0.0073
c	Normal	Linear regression, comparison of slopes	Saline: 0.1837, ethanol: 0.4301
d	Normal	Unpaired *t* test	0.0203
e	Unequal variance (epoch violates Mauchly’s)	rmANOVA with Greenhouse–Geisser correction	Main effect of day: 0.6405 Main effect of exposure: 0.8774 Interaction: 0.0204
f	Normal	rmANOVA	Main effect of tethering: 0.2603 Main effect of exposure: 0.0771 Interaction: 0.0505
g	Normal	rmANOVA	Main effect of time point: 0.0740 Main effect of exposure: 0.0015 Interaction: 0.0004
h	Unequal variance (epoch violates Mauchly’s)	Mixed effect analysis with Greenhouse–Geisser correction	95% CI: −11.50 to 45.05
i	Normal	Linear regression	0.03255
j	Normal	rmANOVA	Main effect of exposure: 0.0037 Main effect of bin: 0.1262 Interaction: 0.2576
k	Normal	rmANOVA	Main effect of exposure: 0.0001 Main effect of bin: 0.2355 Interaction: 0.0046
l	Normal	Unpaired *t* test	0.3682
m	Unequal variance (epoch violates Mauchly’s)	rmANOVA with Greenhouse–Geisser correction	Main effect of day: 0.4277 Main effect of virus: 0.0353 Interaction: 0.0360
n	Normal	Linear regression	GFP: 0.5142, ArchT: 0.0007
o	Normal	Linear regression	GFP: 0.0559, ArchT: 0.1162
p	Normal	rmANOVA	Main effect of virus: 0.2389 Main effect of pairing: 0.0382 Interaction: 0.0767
q	Normal	rmANOVA	Main effect of virus: 0.1193 Main effect of pairing: 0.0204 Interaction: 0.0431
r	Normal	One-sample *t* test	GFP: 0.0785, ArchT: 0.0091
s	Normal	One-sample *t* test	GFP: 0.1253, ArchT: 0.1069
t	Normal	rmANOVA	Main effect of virus: 0.3189 Main effect of pairing: 0.4214 Interaction: 0.0963
u	Normal	rmANOVA	Main effect of virus: 0.0837 Main effect of pairing: 0.0069 Interaction: 0.1129
v	Normal	Unpaired *t* test	0.0013
w	Unequal variance (epoch violates Mauchly’s)	rmANOVA with Greenhouse–Geisser correction	Main effect of day: 0.4270 Main effect of virus: 0.0266 Interaction: 0.0237
x	Normal	Linear regression	GFP: 0.0613, ArchT: 0.1053
y	Normal	Linear regression	GFP: 0.2710, ArchT: 0.0261
z	Normal	Mixed effect analysis	95% CI for virus: −12.44 to 16.73 95% CI for pairing: −19.74 to 0.8410 95% CI for interaction: −30.53 to 10.62 and −10.62 to 30.53
aa	Normal	Mixed effect analysis	95% CI for virus: −1.148 to 0.7127 95% CI for pairing: −1.056 to −0.3407 95% CI for interaction: −0.4423 to 0.9887 and −0.9887 to 0.4423
bb	Normal	One-sample *t* test	GFP: 0.1625, ArchT: 0.1814
cc	Normal	One-sample *t* test	GFP: 0.2774, ArchT: 0.3735
dd	Normal	Mixed effect analysis	95% CI for virus: 1.037 to 18.36 95% CI for pairing: 11.59 to 24.45 95% CI for interaction: −1.029 to 24.68 and −24.68 to 1.029
ee	Normal	Mixed effect analysis	95% CI for virus: −20.17 to 20.89 95% CI for pairing: 16.53 to 40.68 95% CI for interaction: −11.39 to 36.91 and −36.91 to 11.39
ff	Normal	One-way ANOVA	0.167
gg	Unequal variance (epoch violates Mauchly’s)	rmANOVA with Greenhouse–Geisser correction	Main effect of day: 0.2928 Main effect of virus: 0.0878 Interaction: 0.0356
hh	Normal	One-way ANOVA	0.0254
ii	Normal	Unpaired *t* test	0.0043

## Results

### Low-dose ethanol exposure impacts sucrose reward motivation

**Figure 1.**
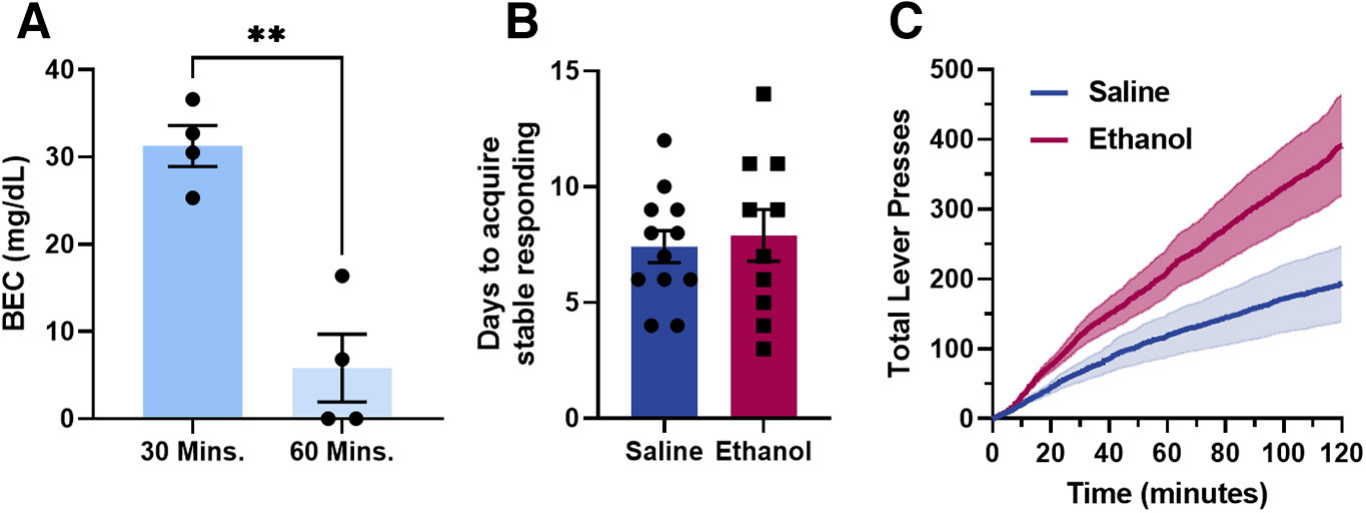
Blood ethanol concentrations (BECs) and behavioral effects of low-dose ethanol exposure. ***A***, Blood ethanol concentrations were elevated at 30 min following injection of low-dose ethanol (0.5 g/kg, i.p.). At 60 min, BECs were not different from baseline. ***B***, Daily exposure to low-dose ethanol daily following training did not impact the number of days it takes to acquire stable responding on an FR1 schedule for a sucrose reward. ***C***, Ethanol-exposed male mice exhibited sustained, accelerated responding during the PR test as compared with saline-exposed mice (**p < 0.01; for BEC data, *n* = 4 per time point; saline *n* = 12; ethanol *n* = 10; error bars represent SEM).

To determine blood ethanol concentrations following low-dose ethanol administration via intraperitoneal injection, bloods were collected and BEC was measured at 30 and 60 min after an injection with low-dose ethanol in separate cohorts of mice (Fig. 1*A*). BECs were significantly lower at 60 min as compared with 30 min after administration (unpaired *t* test, two-tailed, *t* = 5.611, df = 6, *p* = 0.0014). Levels at 60 min were not significantly different from zero (one-sample *t* test vs 0, *t*_(3)_ =1.495, *p* = 0.2318), suggesting that 0.5 g/kg ethanol is mostly cleared at 60 min postinjection.

Previous findings have indicated that acute postlearning exposure to ethanol can facilitate learning (Alkana and Parker, 1979; Tyson and Schirmuly, 1994). To determine whether low-dose ethanol exposure facilitated acquisition of sucrose self-administration, the number of days required to meet acquisition criteria in mice exposed to saline or ethanol (0.5 g/kg, i.p.) 1 h after training was determined. Exposure to low-dose ethanol did not impact the number of days it took for the mice to acquire stable responding (unpaired *t* test, two-tailed, *t* = 0.3829, df = 20, *p* = 0.7058; Fig. 1*B*), suggesting that chronic or repeated exposure to ethanol post-training did not promote reward learning.

Recently published findings from the lab demonstrated that a history of low-dose ethanol exposure increased break points on a PR test as compared with saline exposed controls (Bryant et al., 2022). To compare differences in motivation independent of individual differences in reaching ratio criteria, an additional analysis of this dataset was performed. Total lever presses for each group were measured across the first 2-h block of PR testing (Fig. 1*C*). A comparison of slopes from linear regressions of the cumulative press data revealed that ethanol mice escalated presses at a more rapid rate than saline controls (*F*_(1,172796)_ = 9193, *p* < 0.0001).

### vHPC activity changes across a PR session

**Figure 2.**
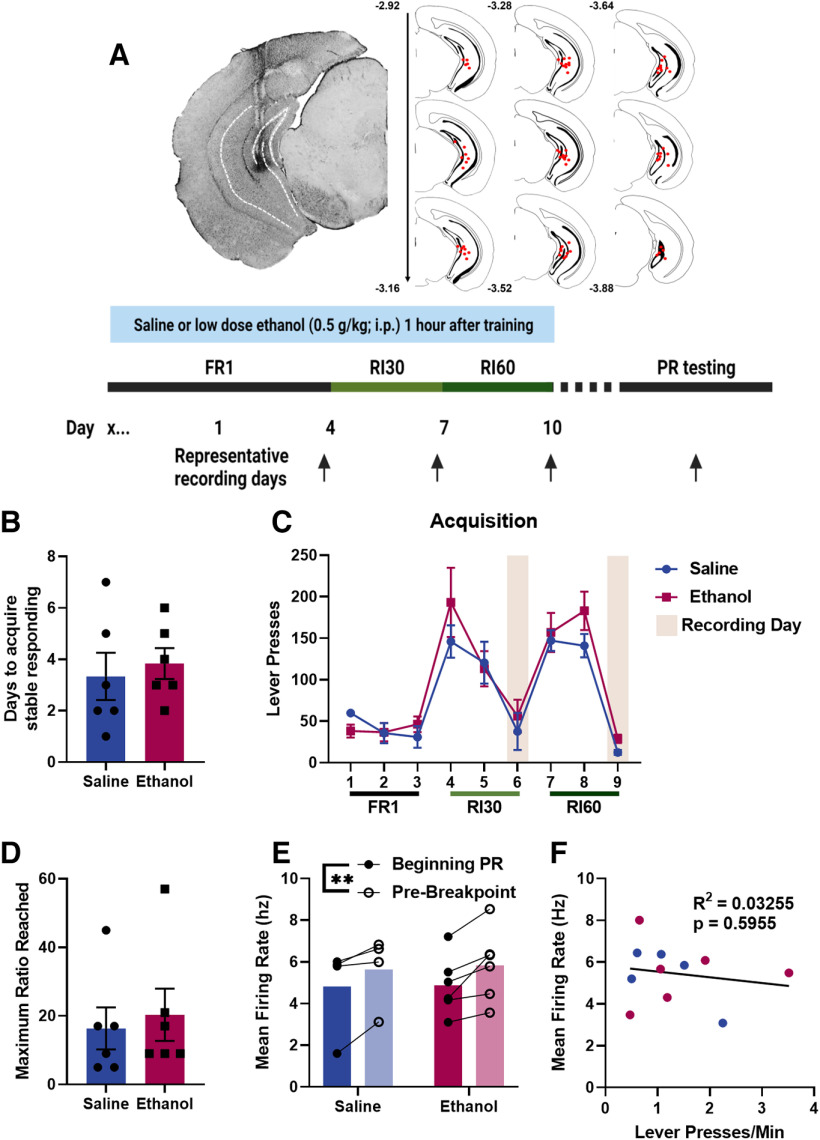
vHPC firing rates during progressive ratio responding. ***A***, A timeline of the behavioral experiment and MEA placements in the vHPC. There was no effect of ethanol on days to acquire stable responding (***B***), basal reward seeking (***C***), or break points during the PR test (***D***). ***E***, vHPC mean firing rates were higher immediately before reaching the break point than the start of the session, independent of ethanol exposure condition. ***F***, vHPC mean firing rates did not correlate with response rates (**p < 0.01; training: saline *n* = 6, ethanol *n* = 6; testing: saline *n* = 4, ethanol *n* = 6; error bars represent SEM).

To determine how vHPC activity was impacted during behavior by a history of post-training, low-dose ethanol exposure, a subset of mice was implanted with a multielectrode array targeting the vHPC and neural activity was recorded during PR testing (Fig. 2*A*). Low-dose ethanol exposure had no impact on either days to acquire stable responding (unpaired *t* test, two-tailed, *t* = 0.4554, df = 10, *p* = 0.6586; Fig. 2*B*) or on reward seeking across training (two-way rmANOVA, main effect of exposure *F*_(1,10)_ = 1.196, *p* = 0.2998; Fig. 2*C*) as compared with saline mice, replicating previous findings (Bryant et al., 2022).

There was a main effect of training day on reward seeking (rmANOVA, Greenhouse-Geisser corrected, *F*_(3.157,31.57)_ = 24.93, *p* < 0.0001). *Post hoc* analyses revealed a significant escalation in responding from the first day of FR1 training to the first days of RI30 (Dunnett’s, day 1 vs 4, *p* = 0.0020) and RI60 (day 1 vs 7, *p* = 0.0001) training, consistent with the escalation in responding often seen when mice are trained on leaner interval-based schedules. As nearly all representative recording sessions for RI30 and RI60 training took place on the final days of each block, responding on those days was reduced in those tethered sessions (days 6 and 9) as compared with the untethered training sessions (days 4, 5, 7, and 8). Responding was also blunted in recording sessions during FR1 training for each mouse, but since these sessions were spaced out across training days and did not occur on the same FR1 training day for all mice, it is not reflected in the overall group averages for FR1 training.

To determine how responding during the PR session in tethered mice compared with previous findings, data from the PR session were compared with previously published findings establishing the effect of low-dose ethanol exposure on PR responding in males (Bryant et al., 2022). Tethering resulted in an overall reduction in break points as compared with nontethered mice (main effect of tethering, two-way ANOVA, *F*_(1,32)_ = 14.59, *p* = 0.0006), consistent to responding on the FR and RI sessions. However, despite this reduction, a main effect of ethanol exposure (two-way ANOVA, *F*_(1,32)_ = 4.321, *p* = 0.0458) was still present and no tethering × ethanol interactions were observed.

To characterize vHPC neural activity changes across the PR test session, vHPC mean firing rate was compared at the beginning of the session (first 2 min of behavior) versus the end, just before the animal’s break point (final 2 min of the last ratio block, determined per animal; Fig. 2*E*). A main effect of time point was observed (rmANOVA, *F*_(1,8)_ = 19.47, *p* = 0.0023), suggesting that vHPC firing rates increased across the PR test session independent of ethanol exposure, as neither a main effect of exposure condition (rmANOVA, *F*_(1,8)_ = 0.01,361, *p* = 0.9100) nor an exposure condition × time point interaction (rmANOVA, *F*_(1,8)_ = 0.1173, *p* = 0.7408) was observed. To determine whether increasing vHPC mean firing rates reflected vHPC tracking of effort, mean firing rates for each ratio block were analyzed. No significant interaction (mixed effects analysis, *F*_(3,16)_ = 1.655, *p* = 0.2166) or main effects (ratio, *F*_(0.9363,4.994)_ = 3.101, *p* = 0.1386; exposure condition, *F*_(1,9)_ = 1.801, *p* = 0.2124) were observed. This suggests that the observed increase in vHPC firing rates was not tied to the overall effort required, but rather the break point of each animal. The mean firing rate for the whole session did not correlate with the response rate for the session (linear regression, *R*^2^ = 0.03,255, *F*_(1,9)_ = 0.3028, *p* = 0.5955), suggesting that differences in vHPC mean firing rates were not related to differences in behavioral activity (Fig. 2*F*).

### Low-dose ethanol exposure modulates vHPC activity during motivated behavior

**Figure 3.**
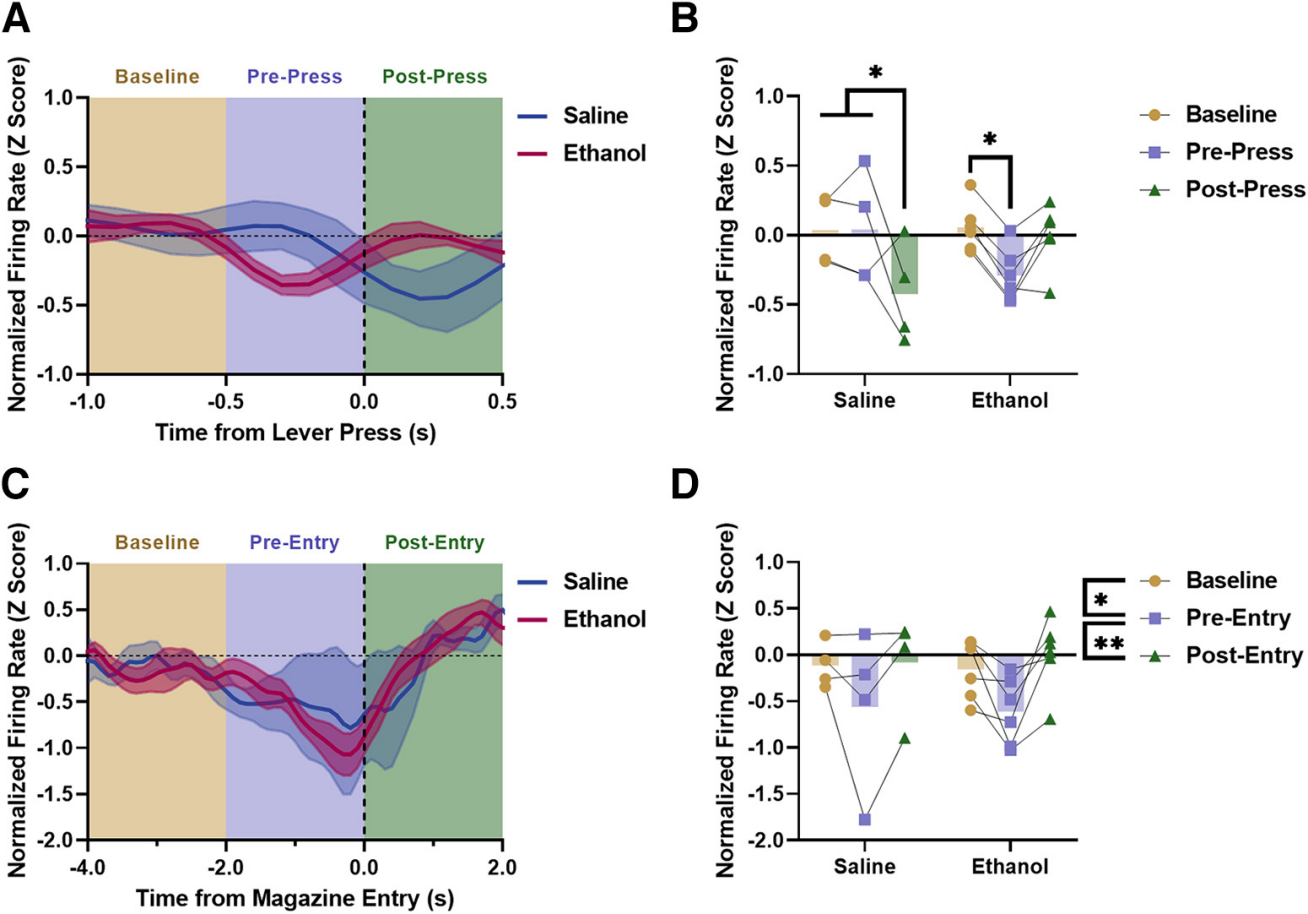
vHPC population activity surrounding discrete behavioral events. ***A***, Normalized vHPC firing rate around lever pressing in ethanol and saline mice. ***B***, vHPC activity was significantly reduced after a lever press in saline controls. In mice with a history of low-dose ethanol exposure, vHPC activity was suppressed before a lever press. ***C***, Normalized vHPC firing rate around magazine entries in ethanol and saline mice. ***D***, vHPC activity was significantly reduced before a magazine entry independent of exposure condition (**p < 0.01, *p < 0.05; saline *n* = 4, ethanol *n* = 6).

In order to investigate the impact of low-dose ethanol exposure on modulation of vHPC activity, vHPC population activity was analyzed around behavioral events important for reward seeking and motivated behavior (Barker et al., 2017). vHPC activity was analyzed surrounding lever press events when the animal was actively seeking the sucrose reward (data has been smoothed using a Gaussian filter for visualization; Fig. 3*A*). Unsmoothed z-scored activity was binned and analyzed during a baseline (−1.0 to −0.5 s), prepress (−0.5 to 0.0 s), and postpress (0.0 to 0.5 s) period based on previous findings and observed activity patterns (Fig. 3*B*). A significant interaction of exposure condition × activity bin was observed (rmANOVA, *F*_(2,16)_ = 7.277, *p* = 0.0057). Šídák’s *post hoc* analyses revealed that saline exposed mice significantly reduced vHPC firing postpress as compared with baseline (*p* = 0.0254) and prepress (*p* = 0.0240), while ethanol exposed mice significantly reduced vHPC firing prepress as compared with baseline (*p* = 0.0381), suggesting that vHPC activity was differentially modulated during reward seeking in the PR as a result of low-dose ethanol exposure. Direct *post hoc* analyses between saline and ethanol for each bin were also completed. No significant effects were found, although there was a trend between ethanol and saline for the postpress bin (Šídák’s *post hoc* analysis, *p* = 0.0595). A trend toward a main effect of activity bin was also observed (rmANOVA, *F*_(2,16)_ = 3.565, *p* = 0.0524).

vHPC activity was then analyzed surrounding entries into the reward delivery magazine (Fig. 3*C*). Only a small percentage of recorded magazine entries during the PR test occurred following reward delivery (3.96%), so the magazine entries analyzed here primarily reflected magazine checking behavior, as opposed to consummatory behavior. Activity was binned and analyzed during a baseline (−4 to −2 s), pre-entry (−2 to 0 s), and postentry (0 to 2 s) period (Fig. 3*D*). Similar to the lever press analyses, these bins were chosen based on previous findings and analyses, as well as on observed activity patterns. A main effect of activity bin was observed (rmANOVA, *F*_(2,16)_ = 7.672, *p* = 0.0046). Šídák’s *post hoc* analyses revealed that vHPC activity was significantly reduced pre-entry as compared with baseline (*p* = 0.0250) and significantly increased postentry as compared with pre-entry (*p* = 0.0061) regardless of ethanol exposure condition, as no main effect of exposure condition was observed (rmANOVA, *F*_(1,8)_ = 1.158e-005, *p* = 0.9974). These findings indicate that vHPC activity was suppressed before a magazine entry during PR testing, and that this suppression was not impacted by a history of low-dose ethanol exposure.

### Inhibition of vHPC during a PR test shifts reward seeking behavior in ethanol-naive male mice

**Figure 4.**
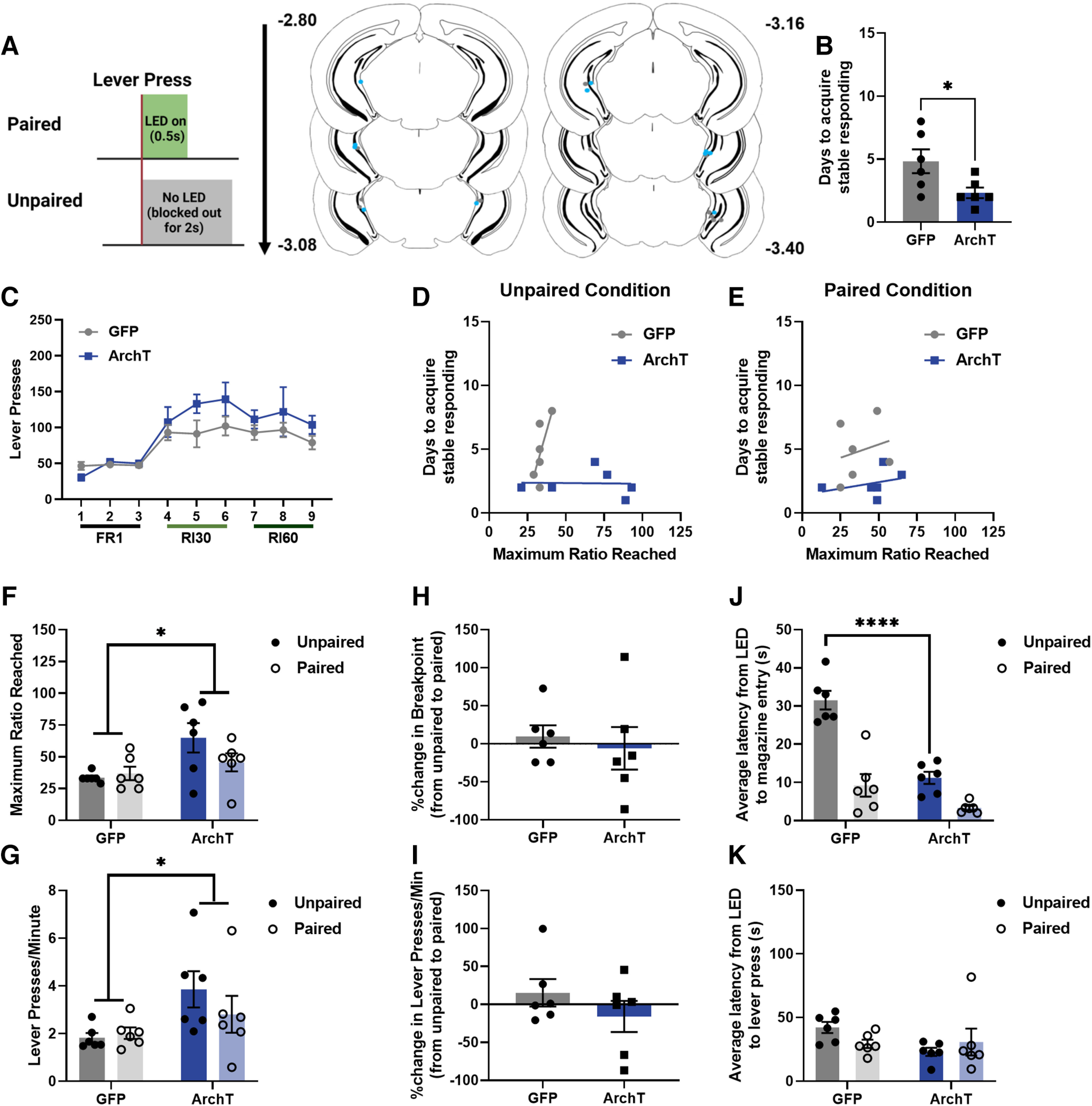
Effects of vHPC optogenetic manipulation in saline-exposed mice. ***A***, Schematic of optogenetic inhibition timing for paired and unpaired groups and optic fiber placements for saline mice. ***B***, ArchT-expressing mice acquired stable responding in fewer days than GFP-expressing mice. ***C***, There was no effect of ArchT expression on basal reward seeking. There was no association between days it took to acquire stable responding and break points during either the unpaired (***D***) or paired (***E***) condition. vHPC inhibition increased break points (***F***) and response rates (***G***) regardless of light pairing, showing that this inhibition is able to increase motivated behavior. Inhibition pairing condition did not affect the percent change in either break points (***H***) or response rates (***I***), again supporting that this is not impacted by whether vHPC inhibition occurs temporally close to a lever press. ***J***, vHPC inhibition significantly reduced latencies to a magazine entry during the unpaired condition, showing that this inhibition is able to drive magazine checking behavior. ***K***, There was no effect of vHPC inhibition on latencies to a lever press, indicating that this is specific to magazine entries (****p < 0.0001, *p < 0.05; GFP *n* = 6, ArchT *n* = 6; error bars represent SEM).

To determine whether vHPC inhibition surrounding a lever press shifted reward seeking behavior, saline-exposed mice were injected with a virus expressing either an inhibitory opsin (ArchT) or control fluorescent protein (GFP) under control of the CaMKIIa promoter and implanted with an optic fiber in the vHPC (Fig. 4*A*). ArchT-expressing mice took significantly fewer days to acquire stable responding on the FR1 schedule than GFP-expressing mice (two-tailed Welch’s unpaired *t* test, *t* = 2.414, df = 6.912, *p* = 0.0469; Fig. 4*B*). For basal reward seeking behavior (Fig. 4*C*) there was neither an interaction of day × virus (rmANOVA, *F*_(8,80)_ = 1.003, *p* = 0.4408) nor a main effect of virus expression (*F*_(1,10)_ = 2.491, *p* = 0.1456) observed. A main effect of day was observed (*F*_(2.911,29.11)_ = 11.90, *p* < 0.0001) such that mice significantly escalated responding by the final day of training as compared with the first (Dunnett’s *post hoc* analysis, day 1 vs 9, *p* = 0.0009). To determine whether these observed differences in training were associated with any differences in break points on the PR test, correlational analyses were performed. There was no correlation between days it took to acquire stable responding and break points during the unpaired condition for either GFP-expressing (linear regression, *R*^2^ = 0.5142, *F*_(1,4)_ = 4.235, *p* = 0.1087) or ArchT-expressing (*R*^2^ = 0.0007, *F*_(1,4)_ = 0.0030, *p* = 0.9591) mice (Fig. 4*D*). There was also no correlation between days it took to acquire stable responding and break points during the paired condition for either GFP-expressing (linear regression, *R*^2^ = 0.0559, *F*_(1,4)_ = 0.2368, *p* = 0.6520) or ArchT-expressing (*R*^2^ = 0.1162, *F*_(1,4)_ = 0.5260, *p* = 0.5085) mice (Fig. 4*E*). Together, these findings confirm that individual or group differences in acquisition or basal reward seeking did not relate to differences in PR testing, which we have also observed previously (Bryant et al., 2022).

To investigate whether the precise epoch of vHPC inhibition relative to a lever press modulated changes in reward seeking behavior, male mice were tested on the PR and the vHPC was inhibited either following a lever press (paired condition) or blocked out from surrounding a lever press (unpaired condition). vHPC inhibition increased the maximum ratio reached (e.g., break point; rmANOVA, main effect of virus, *F*_(1,10)_ = 8.780, *p* = 0.0142; Fig. 4*F*) regardless of LED pairing condition (no pairing × virus interaction, *F*_(1,10)_ = 2.051, *p* = 0.1826; no main effect of pairing, *F*_(1,10)_ = 1.022, *p* = 0.3359). vHPC inhibition also increased overall response rates during the PR test (rmANOVA, main effect of virus, *F*_(1,10)_ = 6.104, *p* = 0.0331; Fig. 4*G*) regardless of LED pairing condition (no pairing × virus interaction, *F*_(1,10)_ = 1.239, *p* = 0.2918; no main effect of pairing, *F*_(1,10)_ = 0.5874, *p* = 0.4611). The percent change from the unpaired to paired condition for both the maximum ratio reached and response rates during the PR test was also analyzed for each animal. There were no significant differences observed either between groups (unpaired *t* test, *t* = 0.4935, df = 10, *p* = 0.6326) or compared with baseline (one-sample *t* test vs 0, GFP, *t* = 0.6524, df = 5, *p* = 0.5429; ArchT, *t* = 0.2144, df = 5, *p* = 0.8387) in percent change of break points from the unpaired to paired condition (Fig. 4*H*). There were also no significant differences observed either between groups (unpaired *t* test, *t* = 1.140, df = 10, *p* = 0.2808) or compared with baseline (one-sample *t* test vs 0, GFP, *t* = 0.8464, df = 5, *p* = 0.4359; ArchT, *t* = 0.7736, df = 5, *p* = 0.4741) in percent change of response rates from the unpaired to paired condition (Fig. 4*I*). These findings show that brief, discrete vHPC inhibition increased break points and response rates on the PR independent of whether this inhibition occurred proximal to reward seeking behavior.

To assess whether vHPC inhibition initiated reward seeking or checking behavior, the latency from the LED light on to either a magazine entry or lever press was measured. For latency from LED to a magazine entry, a significant virus × pairing interaction was observed (rmANOVA, *F*_(1,10)_ = 34.54, *p* = 0.0002; Fig. 4*J*) with Šídák’s *post hoc* analysis revealing a significant reduction in latencies in the ArchT group as compared with the GFP group for the unpaired (*p* < 0.0001), but not paired (*p* = 0.1170), condition. Main effects of virus (*F*_(1,10)_ = 23.54, *p* = 0.0007) and pairing (*F*_(1,10)_ = 151.2) were also observed. For latency from LED to a lever press, no significant interactions (rmANOVA, *F*_(1,10)_ = 2.663, *p* = 0.1338; Fig. 4*K*) or main effects (virus, *F*_(1,10)_ = 2.247, *p* = 0.1648; pairing, *F*_(1,10)_ = 0.1637, *p* = 0.6943) were observed. These findings reveal a selective role for vHPC inhibition in promoting reward delivery checking and tracking – but not seeking – behavior.

### Inhibition of vHPC during a PR test does not shift reward seeking behavior in ethanol-exposed male mice

**Figure 5.**
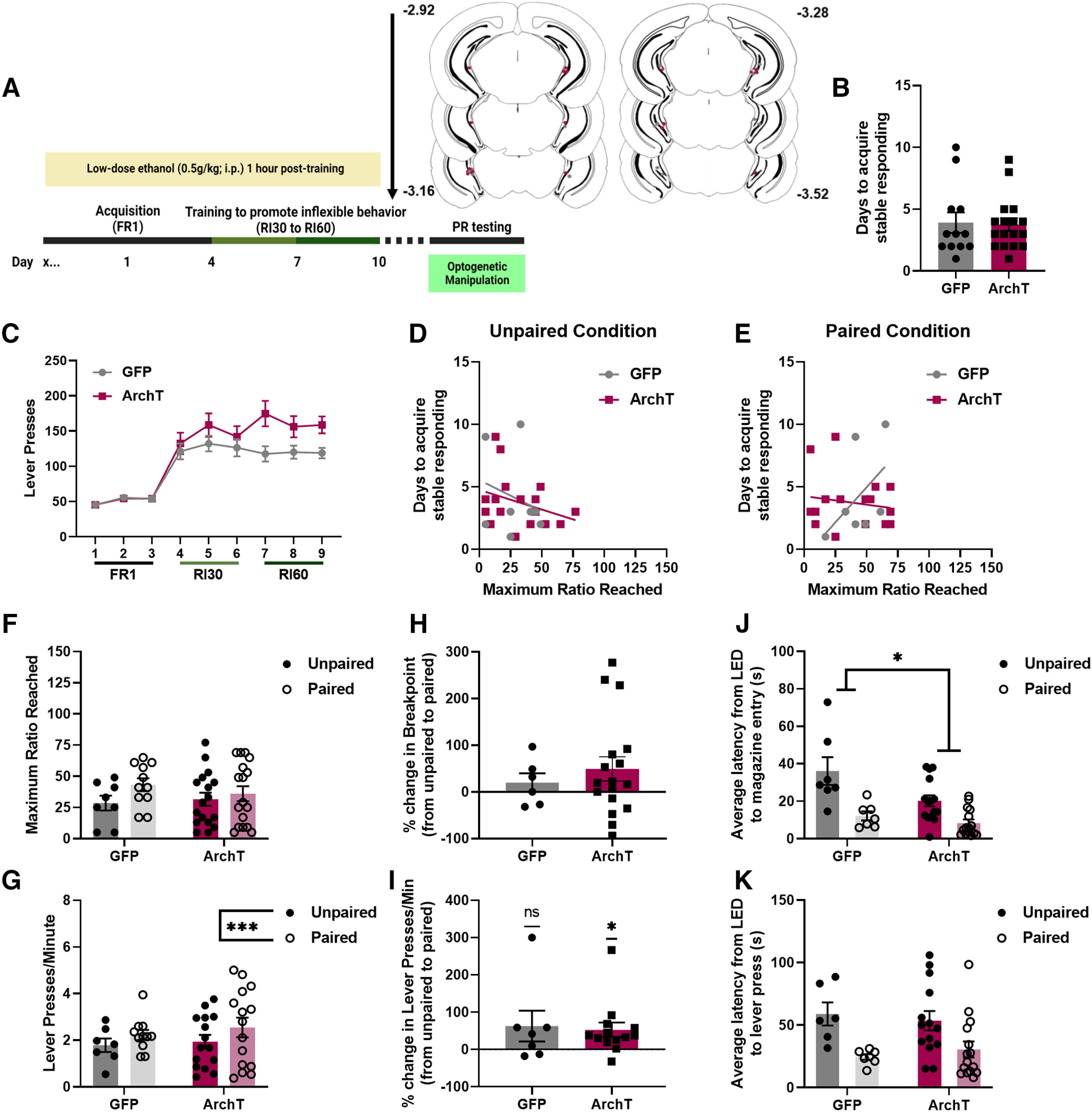
Effects of vHPC optogenetic manipulation in ethanol-exposed mice. ***A***, Experimental timeline and optic fiber placements for ethanol mice. ***B***, There was no effect of ArchT expression on days it took to acquire stable responding. ***C***, ArchT-expressing mice had higher basal reward seeking than GFP-expressing mice. There was no association between days it took to acquire stable responding and break points during either the unpaired (***D***) or paired (***E***) condition. vHPC inhibition had no effect on break points (***F***) and response rates (***G***) regardless of light pairing. There was no effect of inhibition pairing on differences in break points (***H***), but paired vHPC inhibition did increase response rates during the PR test (***I***). ***J***, vHPC inhibition significantly reduced latencies to a magazine entry regardless of pairing condition. ***K***, There was no effect of vHPC inhibition on latencies to a lever press (ns = not significant; ***p < 0.001; *p < 0.05; GFP *n* = 10, ArchT *n* = 16; error bars represent SEM).

To determine whether vHPC activity modulated reward seeking following a history of low-dose ethanol exposure, ethanol-exposed mice were injected with a virus expressing either an inhibitory opsin (ArchT) or control fluorescent protein (GFP) and implanted with an optic fiber in the vHPC same as the saline-exposed mice (Fig. 5*A*). There were no significant differences observed in days to acquire stable responding on the FR1 schedule between GFP-expressing and ArchT-expressing mice (two-tailed Welch’s unpaired *t* test, *t* = 0.1559, df = 19.04, *p* = 0.8777; Fig. 5*B*). For basal reward seeking behavior (Fig. 5*C*), a main effect of virus was observed (rmANOVA, *F*_(1,27)_ = 5.977, *p* = 0.0213) such that ArchT-expressing mice pressed more for sucrose than GFP-expressing mice. A main effect of day was also observed (Greenhouse-Geisser corrected, *F*_(3.751,101.3)_ = 32.05, *p* < 0.0001) such that mice significantly escalated responding by the final day of training as compared with the first (Dunnett’s *post hoc* analysis, day 1 vs 9, *p* < 0.0001). To determine whether these observed differences in training were associated with any differences in break points on the PR test, correlational analyses were performed. There was no correlation between days it took to acquire stable responding and break points during the unpaired condition for either GFP-expressing (linear regression, *R*^2^ = 0.0613, *F*_(1,6)_ = 0.3916, *p* = 0.5545) or ArchT-expressing (*R*^2^ = 0.1053, *F*_(1,15)_ = 1.765, *p* = 0.2039) mice (Fig. 5*D*). There was also no correlation between days it took to acquire stable responding and break points during the paired condition for either GFP-expressing (linear regression, *R*^2^ = 0.2710, *F*_(1,6)_ = 2.231, *p* = 0.1859) or ArchT-expressing (*R*^2^ = 0.0261, *F*_(1,15)_ = 0.4021, *p* = 0.5356) mice (Fig. 5*E*). This is again consistent with our previous findings that any differences in acquisition or basal reward seeking did not relate to differences in PR testing.

To determine whether inhibiting vHPC following reward seeking behavior – the time point when vHPC activity was reduced during motivated behavior in ethanol-naive mice – shifted reward seeking behavior, ethanol-exposed male mice were tested on the PR and the vHPC was inhibited either following a lever press (paired condition) or blocked out from surrounding a lever press (unpaired condition) as described above. vHPC inhibition had no effect on the maximum ratio reached (e.g., break point) regardless of inhibition pairing condition (mixed-effects analysis, no virus × pairing interaction, *F*_(1,23)_ = 1.002, *p* = 0.3273; Fig. 5*F*). vHPC inhibition also had no effect on overall response rates during the PR test (mixed-effects analysis, no virus × pairing interaction, *F*_(1,18)_ = 0.6435, *p* = 0.4329; Fig. 5*G*) but there was a main effect of LED pairing condition (main effect of pairing, *F*_(1,18)_ = 16.83, *p* = 0.0007). The percent change from the unpaired to paired condition for both the maximum ratio reached and response rates during the PR test was also analyzed for each animal. There were no significant differences observed either between groups (unpaired *t* test, *t* = 0.6365, df = 21, *p* = 0.0934) or compared with baseline (one-sample *t* test vs 0, GFP, *t* = 0.9851, df = 5, *p* = 0.3698; ArchT, *t* = 1.883, df = 16, *p* = 0.0780) in percent change of break points from the unpaired to paired condition (Fig. 5*H*). While no significant differences observed between groups (unpaired *t* test, *t* = 0.2502, df = 18, *p* = 0.8052) only mice expressing ArchT exhibited a significant increase in response rate (one-sample *t* test vs 0, GFP, *t* = 1.517, df = 6, *p* = 0.1799; ArchT, *t* = 2.675, df = 12, *p* = 0.0202; Fig. 5*I*). These findings show that discrete vHPC inhibition did not shift break points or response rates on the PR in male mice with a history of low-dose ethanol exposure.

To determine whether vHPC inhibition promoted reward seeking or checking behavior in mice with a history of low-dose ethanol exposure, the latency from the LED light on to either a magazine entry or lever press was measured. For latency from LED to a magazine entry, no significant virus × pairing interaction was observed (mixed-effects analysis, *F*_(1,19)_ = 3.708, *p* = 0.0693; Fig. 5*J*) but there was a main effect of virus (*F*_(1,21)_ = 5.423, *p* = 0.0299) such that vHPC inhibition reduced latencies to a magazine entry regardless of LED pairing. For latency from LED to a lever press, no significant interactions were observed (mixed-effects analysis, *F*_(1,18)_ = 1.232, *p* = 0.2817; Fig. 5*K*) but there was a main effect of pairing (*F*_(1,18)_ = 24.77, *p* < 0.0001) such that mice had lower latencies to a lever press from LED during the paired condition. These findings reveal that while vHPC inhibition promoted different behavioral strategies in ethanol-exposed mice, it did not increase overall motivated behavior and reward seeking as was observed in ethanol-naive mice. This suggests that low-dose ethanol exposure produced long-term changes in vHPC regulation of reward seeking.

### Chronic vHPC inhibition during training does not impact reward seeking behavior

**Figure 6.**
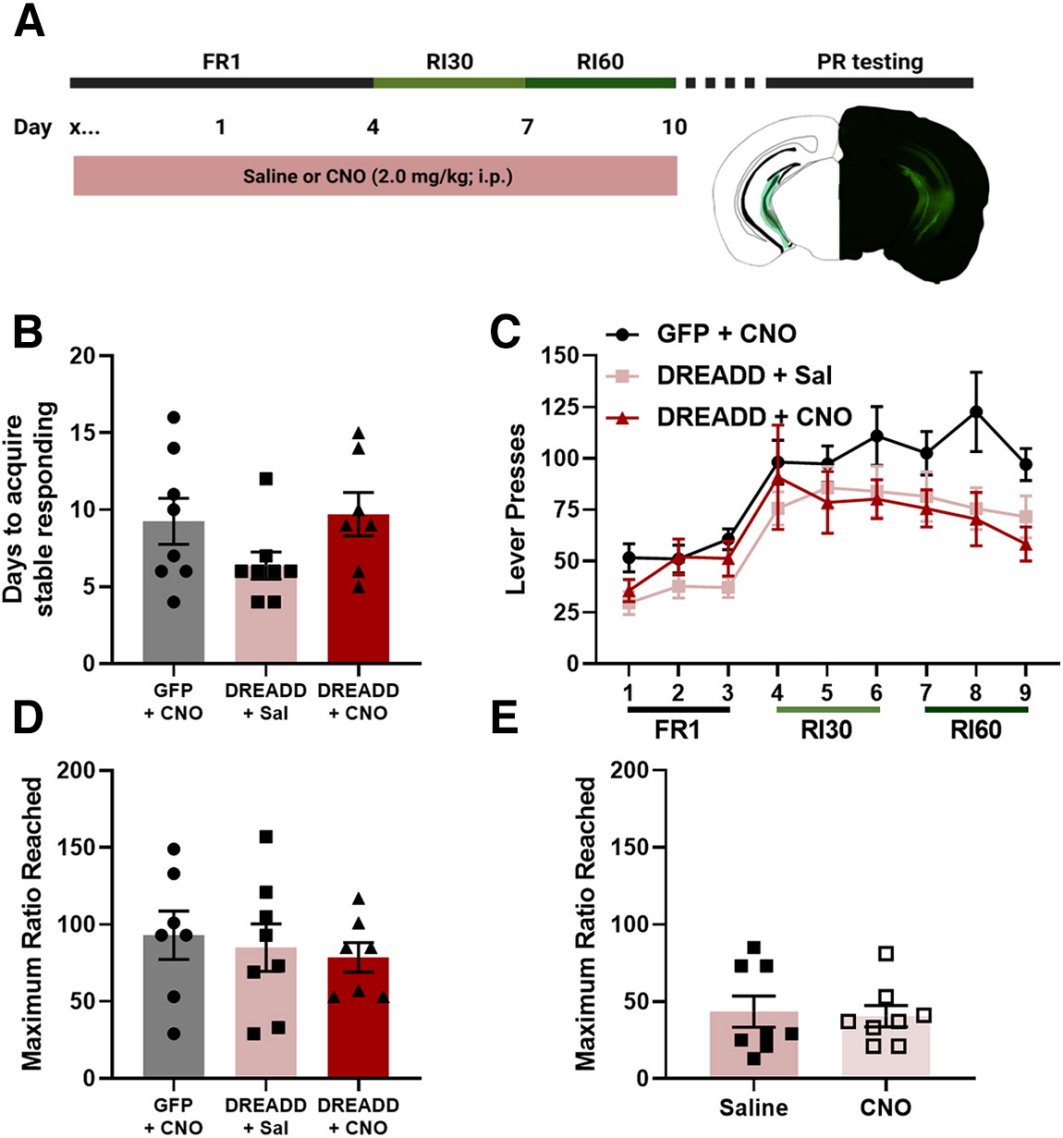
Effects of vHPC chemogenetic manipulation on reward seeking. ***A***, Experimental timeline and representative placements of minimal and maximal virus expression. Chemogenetic vHPC inhibition during task acquisition did not affect the days it took to acquire stable responding (***B***) or basal reward seeking (***C***). Neither a history of vHPC inhibition during acquisition (***D***) nor expression of motivated behavior (***E***) affected break points on the PR test (GFP + CNO *n* = 8, DREADD + Sal *n* = 8, DREADD + CNO *n* = 7; error bars represent SEM).

To determine whether a history of vHPC inhibition throughout training could alter reward seeking behavior, male mice were injected with a virus expressing either a DREADD or GFP, and then were exposed to either CNO or saline throughout training (Fig. 6*A*). There was no effect of vHPC inhibition during training on days it took to acquire stable responding (one-way ANOVA, *F*_(2,20)_ = 2.005, *p* = 0.1609; Fig. 6*B*). For basal reward seeking, there was a main effect of virus (rmANOVA, *F*_(2,20)_ = 3.730, *p* = 0.0420; Fig. 6*C*) such that the GFP + CNO mice had greater responding than both the DREADD + Sal (Tukey’s *post hoc* analysis, *p* < 0.0001) and DREADD + CNO (*p* = 0.0001) mice. There was also a main effect of day (Greenhouse-Geisser corrected, *F*_(3.888,77.76)_ = 17.17, *p* < 0.0001) such that all groups escalated responding on the final day of training as compared with the first (Dunnett’s *post hoc* analysis, day 1 vs 9, *p* < 0.0001). To determine whether there were differences in behavioral strategy selection during training as a result of vHPC inhibition, magazine checking behavior as measured by the percentage of lever presses followed by a magazine entry were analyzed for each training day as described previously (Bryant et al., 2022). There were no significant main effects (rmANOVA, no main effect of day, Greenhouse-Geisser corrected, *F*_(4.044,74.31)_ = 2.043, *p* = 0.0963; no main effect of virus, *F*_(2,21)_ = 0.6792, *p* = 0.5178) or interaction (*F*_(16,147)_ = 0.6998, *p* = 0.7908), suggesting that vHPC inhibition does not shift magazine checking behavior during training.

There was no effect of a history of vHPC inhibition during training on the maximum ratio reached on the PR (one-way ANOVA, *F*_(2,20)_ = 0.8544, *p* = 0.4405; Fig. 6*D*). To determine whether vHPC inhibition during the expression of motivated behavior (i.e., during the PR test) shifted motivated behavior, mice from the DREADD + Saline group were given additional PR test days in which they received injections of CNO or saline 30 min before the PR test. No effect of vHPC chemogenetic inhibition during test on the maximum ratio reached during the PR test was observed (unpaired *t* test, *t* = 0.2464, df = 14, *p* = 0.8089; Fig. 6*E*). These findings show that, in contrast to the effects of discrete vHPC inhibition on behavior observed with optogenetic manipulation, chemogenetic inhibition of the vHPC inhibition during acquisition or expression of motivated behavior does not shift reward seeking.

## Discussion

The current findings demonstrate that chronic low-dose ethanol exposure altered vHPC regulation of reward seeking behavior. Chronic low-dose ethanol exposure increased reward motivation in male mice (Bryant et al., 2022), and these behavioral alterations were accompanied by shifts in vHPC suppression surrounding instrumental actions as compared with ethanol-naive controls. The observed shift in vHPC activity following chronic low-dose ethanol exposure was specific to instrumental action as there were no effects of ethanol on activity surrounding reward checking or collection. Consistent with a role for vHPC activity in regulating reward seeking strategy under control conditions, optogenetic inhibition of the vHPC increased break points in saline-exposed male mice, while this manipulation did not impact behavior in ethanol-exposed mice. Independent of ethanol exposure history, brief inhibition of the vHPC promoted reward checking behavior. This protracted change in a neural correlate of reward-seeking behavior and the effect of low-dose ethanol exposure on vHPC regulation of reward seeking identify long-term neurobehavioral consequences of chronic low-dose ethanol exposure.

This work identifies shifts in vHPC activity surrounding reward seeking as a neural correlate of altered reward motivation following chronic low-dose ethanol exposure. Specifically, in ethanol-naive control mice, reduced vHPC firing rates followed an instrumental action. A history of low-dose ethanol exposure shifted the timing of vHPC activity suppression around lever pressing such that in low-dose ethanol-exposed mice this suppression anticipated the action. In contrast to patterns of vHPC activity surrounding lever presses, suppression of vHPC activity before a magazine entry occurred in both ethanol-exposed and control mice. This replicates findings from Yoshida and colleagues which showed that reward seeking behavior was associated with reductions in vHPC calcium activity (Yoshida et al., 2019, 2021), suggesting that there are population level reductions in vHPC activity during goal-directed actions. Consistent with the idea that suppression of vHPC activity after a lever press is related to goal-directed behavior, the shift observed in ethanol-exposed mice could reflect a reliance on more habitual or inflexible response strategies. Such shifts in neural activity as a result of differences in response strategy selection have been observed in other cortical regions that regulate reward seeking (Barker et al., 2017), and modulating vHPC circuit activity can shift response selection (Barker et al., 2019).

Optogenetic inhibition of the vHPC increased break points in saline-exposed mice, regardless of whether the inhibition was paired to a lever press or not. This is consistent with previous findings that optogenetic inhibition of the vHPC increased break points on a PR test (Yoshida et al., 2019). However, in the current findings, brief vHPC inhibition (0.5 s) did not shift break points in mice with a history of ethanol exposure. This, combined with our findings showing shifts in vHPC activity, suggests that chronic low-dose ethanol altered how vHPC regulates reward seeking such that transient vHPC inhibition is no longer able to increase break points on the PR.

In both ethanol-naive and ethanol-exposed mice, optogenetic inhibition reduced latencies to enter the reward magazine in ArchT-expressing mice compared with light delivery in GFP-expressing controls, suggesting that the inhibition itself promotes magazine checking. However, there was a significant interaction only in ethanol-naive mice, such that ArchT-expressing mice exhibited significantly lower latencies to enter the reward magazine specifically in the unpaired inhibition condition. Importantly, in the unpaired condition the inhibition is no longer tied to, and is indeed explicitly blocked out from, lever pressing, so this represents a time point when the mouse is likely not engaged in the task. Thus, while vHPC inhibition promoted similar changes in reward tracking in both ethanol-naive and ethanol-exposed mice, these changes were attenuated and did not translate to higher break points or depend on task engagement in ethanol-exposed mice.

The effect of vHPC inhibition on reward checking and motivated behavior is consistent with the role of vHPC in encoding reward-related contextual cues and environmental contexts (Corbit and Balleine, 2000; Ito et al., 2008; Sweeney and Yang, 2015; Schumacher et al., 2016; Riaz et al., 2017) and in tracking goal-related information (Kutlu and Gould, 2016; Gauthier and Tank, 2018). It has been theorized that the vHPC is a comparator of conflicting stimuli that ensures appropriate behavioral strategy selection, such that general suppression of vHPC activity is necessary for the performance of discrete actions (McNaughton and Gray, 2000). That vHPC activity is generally suppressed, rather than increased, around discrete behavioral events is consistent with the hypothesis that suppression of vHPC activity is required for behavioral performance. Both saline-exposed and ethanol-exposed mice exhibited similar patterns of vHPC suppression leading up to a magazine entry. That optogenetic inhibition of the vHPC was able to promote magazine checking in both saline-exposed and ethanol-exposed mice supports that this observed suppression of vHPC was driving a magazine entry. Our recently published findings showed that reward tracking behavior as measured by magazine checking is correlated with break points in ethanol-exposed male mice such that reward tracking was lower in mice that reached higher ratio break points (Bryant et al., 2022). This indicates that ethanol exposure is inducing changes in both vHPC activity and in the ability of modulating vHPC activity to modify behavior. This may implicate vHPC as a secondary structure in the regulation of reward seeking in mice with a history of chronic low-dose ethanol, thus suggesting a dissociation in the neural control of reward motivation in the chronic ethanol-exposed state.

Indeed, the ethanol-driven shifts in vHPC neural modulation may reflect changes in discrete circuits or populations that regulate reward seeking (Seib et al., 2021; Sánchez-Bellot et al., 2022). The vHPC sends robust excitatory innervation to regions that have been extensively studied in reward, including the nucleus accumbens, prefrontal cortex, and basolateral amygdala (French and Totterdell, 2003; Ishikawa and Nakamura, 2006; Britt et al., 2012; Kim bin and Cho, 2017; Yang and Wang, 2017; Beyeler et al., 2018; Liu and Carter, 2018). It is known that the role of vHPC in reward seeking depends on activation and suppression of specific circuits (Bryant and Barker, 2020), and identification of these discrete substrates of reward motivation will help advance our understanding of the neurobiology of motivated behavior and the consequences of low-dose ethanol exposure. One note is that most vHPC outputs, including those listed above, are through the vCA1 subregion (Bryant and Barker, 2020; Gergues et al., 2020), while the current findings were primarily recording from and manipulating the vCA3 subregion. The vCA3 does have some long-range targets that would be notable for reward seeking, such as the dorsal lateral striatum (DLS) and lateral septum (LS; Besnard et al., 2020), but it acts primarily as a modulator and driver of vCA1 output through robust intrahippocampal projections (Witter, 2007; Fanselow and Dong, 2010). Thus, primarily vCA3 modulation as presented here would still produce changes in vCA1 activity and its downstream targets.

The current findings indicate that vHPC neural activity is increased immediately before reaching the break point as compared with the beginning of the PR session. However, vHPC activity was not tied to the level of overall effort required, as vHPC firing rates were increased before the break point for nearly all mice, regardless of the actual ratio reached. Further, while these data point toward a relationship between vHPC firing rates and break points, the structure of the behavioral assay in the tethered animals precluded further investigation of this possibility. In future studies, shifting the PR schedule and break point definition may be appropriate to accommodate baseline differences in responding frequency as a result of task design. However, general vHPC inhibition during the PR test using chemogenetics was not sufficient to shift PR break points, while the small, online vHPC inhibition using optogenetics was. This, in addition to the lack of effect of chemogenetic inhibition during acquisition and the lack of a relationship between responding during training and break points on the PR, suggests that it is not general vHPC activity, but instead transient changes in vHPC activity surrounding specific behavioral events that is important for reward seeking.

Together, these findings demonstrate that chronic low-dose ethanol exposure produces long-lasting neurobehavioral effects. These alterations are accompanied by ethanol-induced perturbations in vHPC neural activity modulation during reward seeking which disrupts vHPC regulation of reward seeking. These novel findings have important implications for the investigation of chronic low-dose ethanol effects on the brain and behavior and demonstrate that even low doses produce long-lasting effects on neurobiology. Identification of the neural substrates underlying aberrant motivation following chronic low-dose ethanol exposure, which more closely reflects the drinking patterns of a substantial number of people, is expected to inform the targeting and development of novel pharmacotherapeutics to help prevent the transition from casual drinking patterns to drinking behavior consistent with alcohol use disorders.

## Synthesis

Reviewing Editor: Tod E Kippin, University of California Berkeley Decisions are customarily a result of the Reviewing Editor and the peer reviewers coming together and discussing their recommendations until a consensus is reached. When revisions are invited, a fact-based synthesis statement explaining their decision and outlining what is needed to prepare a revision will be listed below. The following reviewer(s) agreed to reveal their identity: Foster Olive. Note: If this manuscript was transferred from JNeurosci and a decision was made to accept the manuscript without peer review, a brief statement to this effect will instead be what is listed below.

I have received two independent reviews of your manuscript and both reviewers highlighted the many strengths of the manuscript while indicating only a few minor concerns. As such, this study is likely to be suitable for publication but I will need you to respond to all of the reviewer comments (below) and where necessary revise the manuscript before I can make a final decision. I am available to discuss any of the comments if that is helpful during the revision process.

Reviewer 1

The manuscript has been previously reviewed at another journal and revised accordingly. The authors describe studies showing that ventral hippocampal (vHPC) activity is suppressed immediately post-reward during progressive ratio responding for sucrose in ethanol naïve mice, but in mice previously exposed to low dose ethanol vHPC activity anticipates reward seeking. Activity was suppressed in both conditions during reward magazine entry. Utilizing optogenetics, the authors show that inhibition of vHPC increased sucrose seeking only in ethanol naïve mice, and promoted inspection of the reward magazine in both groups. There were no apparent effects of chemogenetic modulation of the vHPC. Overall, this is a good study and a well-written manuscript that provides new information about the role of vHPC activity in tracking and regulating reward seeking following low dose ethanol exposure.

There are a few minor issues that need to be addressed:

- Line 71 - when describing the tracing vector, it would be better to add subscript “rg” after AAV to delineate that it is a retrograde preferring serotype.

- Remove hyphen between “Med” and “Associates”

- Ethanol exposure section starting on line 123 - please state the concentration of alcohol (i.e., 20% v/v) used for i.p. administration

- Line 134 - change “the bloods” to “the blood samples”

- Line 171 - it is unclear why the authors chose to stain tissue for astrocytes (GFAP) for confirmation of wire placement. Is there something specific about astrocytes the authors wish to convey, as opposed to staining tissue with Nissl or other histology stain? It is an acceptable method, just needs a brief rationale.

- There is no mention of the chemogenetics findings in the abstract

- Please place group sizes (n’s) either in the figure legends or the figures themselves

- Figures 2A, 4A and 5A - please enlarge the photograph and/or diagrams of the hippocampus so that placements is more legible. Also, in Fig. 4A, please use a color other than blue to delineate placements, as it is difficult to visualize against the black outlines.

Reviewer 2

I have read the transferred article concerning the role for the vHPC in modulating reward (sucrose) seeking behavior. I have only a few comments.

1. An explanation as to why male subjects were employed exclusively should be included as the expectation is that studies employ both male and female subjects.

2. line 559. For the paragraph beginning with “This”, it is not clear what “this” is. I can understand why the authors started a new paragraph and combining the two paragraphs would create a very long paragraph but the transition between paragraphs should be made more clear.

## Author Response

Synthesis Statement for Author (Required):

I have received two independent reviews of your manuscript and both reviewers highlighted the many strengths of the manuscript while indicating only a few minor concerns. As such, this study is likely to be suitable for publication but I will need you to respond to all of the reviewer comments (below) and where necessary revise the manuscript before I can make a final decision. I am available to discuss any of the comments if that is helpful during the revision process.

Reviewer 1

The manuscript has been previously reviewed at another journal and revised accordingly. The authors describe studies showing that ventral hippocampal (vHPC) activity is suppressed immediately post-reward during progressive ratio responding for sucrose in ethanol naïve mice, but in mice previously exposed to low dose ethanol vHPC activity anticipates reward seeking. Activity was suppressed in both conditions during reward magazine entry. Utilizing optogenetics, the authors show that inhibition of vHPC increased sucrose seeking only in ethanol naïve mice, and promoted inspection of the reward magazine in both groups. There were no apparent effects of chemogenetic modulation of the vHPC. Overall, this is a good study and a well-written manuscript that provides new information about the role of vHPC activity in tracking and regulating reward seeking following low dose ethanol exposure. There are a few minor issues that need to be addressed: - Line 71 - when describing the tracing vector, it would be better to add subscript “rg” after AAV to delineate that it is a retrograde preferring serotype. This has been added to the manuscript so that is now reads “pAAVrg-hSyn-EGFP” (now line 73). 2

- Remove hyphen between “Med” and “Associates” This change has been made (line 82).

- Ethanol exposure section starting on line 123 - please state the concentration of alcohol (i.e., 20% v/v) used for i.p. administration It was 7% v/v. This information has been added to the manuscript (now line 132).

- Line 134 - change “the bloods” to “the blood samples” This change has been made (now line 134).

- Line 171 - it is unclear why the authors chose to stain tissue for astrocytes (GFAP) for confirmation of wire placement. Is there something specific about astrocytes the authors wish to convey, as opposed to staining tissue with Nissl or other histology stain? It is an acceptable method, just needs a brief rationale. We agree with the reviewer that the justification for the use of GFAP staining was not clear. Other staining methods, like Nissl, would not allow for the detection of the wire track tips, without first causing tissue ablation at the tips, due to the thinness of the wires. As a glial scar progressively forms around the wires, staining with GFAP allows for the visualization of the tracks through visualization of the reactive astrocyte response, without the need for tissue ablation. A note clarifying this point has been added to the manuscript (lines 170-175).

- There is no mention of the chemogenetics findings in the abstract A sentence on the findings has now been included in the abstract (lines 16-18). “There was no effect of chemogenetic inhibition of the vHPC either during training or testing on sucrose reward motivation.”

- Please place group sizes (n’s) either in the figure legends or the figures themselves When possible, individual data points, which reflect the number of animals per group have been included for all graphs. Group n’s have been added at the end of each figure legend.

- Figures 2A, 4A and 5A - please enlarge the photograph and/or diagrams of the hippocampus so that placements is more legible. Also, in Fig. 4A, please use a color other than blue to delineate placements, as it is difficult to visualize against the black outlines. The placement images for each of these figures have been enlarged and should reflect the size of individual data points on the graphs. For Fig. 4A, a brighter blue with greater contrast has been used to improve visibility in addition to the larger sizing.

Reviewer 2 I have read the transferred article concerning the role for the vHPC in modulating reward (sucrose) seeking behavior. I have only a few comments. 3

1. An explanation as to why male subjects were employed exclusively should be included as the expectation is that studies employ both male and female subjects. The goal of this study was to investigate the neural correlates underlying low-dose ethanol-induced disruptions in reward seeking, which were only observed in male, but not female, mice (Bryant et al., 2022). Thus, only male mice were used in this study. This context and justification have been added to the methods in the “subjects” section (lines 68 - 70).

2. line 559. For the paragraph beginning with “This”, it is not clear what “this” is. I can understand why the authors started a new paragraph and combining the two paragraphs would create a very long paragraph but the transition between paragraphs should be made more clear.

This has been clarified so that it now reads “The effect of vHPC inhibition on reward checking and motivated behavior is consistent with [...]” (now line 551).
